# Substituting Solid Fossil Fuels with Torrefied Timber Products

**DOI:** 10.3390/ma16247569

**Published:** 2023-12-08

**Authors:** Jan Malaťák, Martin Jankovský, Jitka Malaťáková, Jan Velebil, Arkadiusz Gendek, Monika Aniszewska

**Affiliations:** 1 Department of Technological Equipment of Buildings, Faculty of Engineering, Czech University of Life Sciences Prague, Kamýcká 129, 16500 Prague, Czech Republic; malatak@tf.czu.cz; 2 Department of Forest Technologies and Construction, Faculty of Forestry and Wood Sciences, Czech University of Life Sciences Prague, Kamýcká 129, 16500 Prague, Czech Republic; 3 Department of Economics, Faculty of Economics and Management, Czech University of Life Sciences Prague, Kamýcká 129, 16500 Prague, Czech Republic; malatakova@pef.czu.cz; 4 Department of Biosystems Engineering, Institute of Mechanical Engineering, Warsaw University of Life Sciences—SGGW, Nowoursynowska 164, 02-787 Warsaw, Poland; arkadiusz_gendek@sggw.edu.pl (A.G.); monika_aniszewska@sggw.edu.pl (M.A.)

**Keywords:** renewable fuels, torrefaction, spruce, biochar, economic analysis

## Abstract

As a push towards alternative and renewable resources for heat and power generation, biomass and thermally treated fuels from biomass may be viable options in the upcoming economic reality. This study the verified mass and energy balance of spruce woody biomass after low temperature pyrolysis between 250 and 550 °C. The results showed that low-temperature pyrolysis can yield high-grade biochar suitable for substitution of fossil fuels. Crucially, the net calorific value of biochar processed at 350 °C substantially exceeded that of brown coal. An economic analysis was carried out on the assumption of the current economic reality in the Czech Republic. It was shown that even if the price of the biochar slightly increased, it would still be beneficial to invest in torrefaction technology over paying carbon credits.

## 1. Introduction

Coal is the main fuel used for heat and power production globally, while also substantially contributing to greenhouse gas emissions [1]. China is the largest CO_2_ emitter [2,3]. Other large CO_2_ emitting countries include the USA, India, Australia, Indonesia, Russia, South Africa, Germany, and Poland [4]. According to Demirbas [5], the substitution of the fossil-based fuels remains a great challenge, while Goyal et al. [6] and Gürdil et al. [7] identified pyrolysis technologies that use renewable biological resources as capable of producing viable coal substitutes for heat and power production [8] or chemical processes [9].

Czech energy policy prioritizes viewing alternative or renewable biomass resources as fuels and using them wherever it is technically and economically feasible. This policy prefers the use of timber damaged by large-scale biotic forest disturbances, logging residues, small-diameter wood, and other types of wood not usable in higher added value supply chains as alternative fuels [10]. Šafařík et al. [10] estimated that the total production of local, low-grade wood-based biomass available for energy production will reach 13.47 million tonnes by the year 2036. Furthermore, the need to fulfil these goals is substantiated also by the plan to decarbonize the Czech energy sector [11], i.e., the termination of coal mining for energy production between 2033 and 2038.

One potential treatment of woody biomass that improves its usability in energy production is torrefaction—i.e., controlled carbonization within the 200 and 350 °C temperature range under a non-oxidizing atmosphere [12]. This process decreases the moisture content in the biomass, sterilizes it, and increases its energy density [13]. Wood is thereby transformed into a product with parameters comparable to coal [14], leading to an improved ability to be a substitute for solid fossil fuels [15,16,17,18], while minimizing the need to invest in converting existing combustion plants to enable the use of biomass [19] and also while enabling the financing of the necessary investments through savings of carbon credits traded within the European Union Emission Trading System [20].

According to Jeníček et al. [21], Chen et al. [22], and Gendek et al. [23], torrefied biomass has three main advantages over unprocessed biomass—increased heating value, increased energy density, and improved physical characteristics, such as shape retention [24], homogeneity [23], and hydrophobic behaviour [25]. The heating value may increase from 17 up to 27 MJ kg^−1^ [26] through torrefaction, which is enabled by the decrease in oxygen content [27,28,29]. Simultaneously, hydrogen content decreases [30] and the decrease in the hydroxyl groups in the material contributes to its hydrophobic behaviour [17]. These characteristics lead to an overall decrease in weight and therefore also a decrease in logistics costs and in the need for storage space, and to increased safety while handling [31]. According to Tumuluru et al. [16], further advantages are, especially for herbal biomass, combustion problems in large-scale boilers are greatly diminished, e.g., clogging, sintering, chlorine corrosion, etc. On the other hand, torrefaction leads to the loss of about 10% of the energy accumulated in the biomass when heated to temperatures exceeding 300 °C [32].

Cha et al. [33] reported that torrefaction has substantial potential, its advantage being that it can be used both in large, combined heat and power plants (CHP) and small-scale, mobile cogeneration units, which are already available on the market [31]. Studies show that integration of a torrefaction and cogeneration unit at full loading is beneficial [20,34,35]. The available sources showed that torrefaction has been studied only on selected timber grades, but holistic data on torrefaction of wood-based residues are lacking.

Solid torrefaction products, produced from renewable biological resources, can diversify the use of biomass for energy, and can be considered a carbon neutral fuel [36]. Moreover, a wide range of biomasses can be torrefied [37] and because the product has properties similar to solid fossil fuels [22], the conversion of current coal-burning technologies to using torrefaction products is rather cost-effective, compared to burning thermally unprocessed biomass [38,39], or using gasification technologies [40]. According to Peters et al. [41], co-combustion of biochar with coal brings the most environmental and energy benefits, though, as Ippolito et al. [42] stated, biochar has multiple other uses, such as a soil conditioner, which increases carbon sequestration in soil, among other benefits.

The Central and Eastern European region is, due to its historical preference of coniferous, low-diversity, high-yield forests, substantially threatened by climate change impacts. The largest recent forest disturbances in the region occurred in the Czech Republic because of multiple factors acting on the forest ecosystems. Besides other factors, Czechia experienced low precipitation and high temperatures over the years 2018–2019 [43]. These lowered the natural resilience of trees and simultaneously provided favourable conditions for propagation of the bark beetle (*Ips typographus*), a typical secondary disturbance agent, which targets drought-stressed or otherwise vulnerable Norway spruce trees [44]. Combined with factors that hindered the ability of Czech foresters to act quickly (e.g., the regulatory framework and operational factors), Czechia experienced multiple years when the volume of harvested timber exceeded typical volumes by a factor of two [45]. For example, in the year 2018, the volume of salvage-logged timber exceeded 23 million m^3^ [46], i.e., 90% of the total harvests of 25.69 million m^3^ [47]. The processing of the disturbance is not over, as can be seen from figures for the year 2021, when the volume of harvested timber reached 30.26 million m^3^ of timber, of which, 26.28 million m^3^ was salvage logging. Still, the volume is 5.77 million m^3^ lower than the 2020 harvested timber volume [47]. Besides the large quantities of timber available, disturbances also affected the quality.

Calamity timber has more variability in quality, and a substantial part of the salvage-logged timber cannot be processed otherwise than as a fuel. Biotic disturbance of a tree goes through three stages of attack as evidenced by needle degradation [48,49]. Infested trees are exposed to increasing stress and face physiological changes through interruption of water flow and damage to chloroplasts [48]. This leads to a gradual change in the chemical composition and water content of wood, which causes changes in its spectral characteristics during the attack and the overall degradation of the tree [49]. A further source of input material is small-diameter wood and logging residues, which constitute about 60% of all aboveground biomass [50]. These biomass streams can contribute to reaching the target of fossil fuel substitution as formulated in EU strategic documents.

The aim of this article is to assess the potential of using biotic disturbance-damaged timber for energy through torrefaction. The first step we determine the quality parameters of torrefaction products and identify factors that affect said parameters. Further, we assess the compliance of torrefaction products with the requirements of energy processing technologies and assess the economic feasibility of the conversion of a model coal-based CHP to the use of torrefied wood.

## 2. Materials and Methods

### 2.1. Qualitative Parameters of Solid Products

The timber used in our analyses was collected in 2022 from Vysočina region of the Czech Republic. The spruce samples were taken from wood logs 6–24 months after felling. From these samples, wood chips were made, and partial samples were combined to make an average material sample for the subsequent analyses.

To determine the qualitative parameters, fuel tests were conducted, which included determination of the non-combustible content (moisture, ash content), gross and net calorific values, and the elemental composition. The biomass samples were milled on a Retsch SM 100 mill to a 1 mm particle size. These samples were thermally treated in a LECO TGA 701 programmable furnace (LECO Corporation, St. Joseph, MI, USA) in aluminium-lined crucibles with approximately 3 g of material in at least 10 repetitions over 30 min, with a 20 °C min^−1^ temperature increase in an inert atmosphere. For optimization, five temperatures were chosen—250 °C, 300 °C, 350 °C for low-temperature pyrolysis (i.e., torrefaction), and 450 °C and 550 °C as higher temperatures. The resulting biochars were collected for sample analyses.

The moisture and ash contents were analysed in an automatic thermogravimetric analyser LECO TGA701 (LECO Corporation, St. Joseph, MI, USA). Approximately 1 g of the samples was inserted into ceramic crucibles. Moisture content was analysed at 105 °C to constant mass. Ash content was determined as the residue after incineration of the samples at 550 °C with oxygen to constant mass. Each material was analysed in five repetitions.

The elemental composition of the samples was analysed using a LECO CHN628+S (LECO Corporation, USA) analyser. The device determined the content of carbon (C), hydrogen (H), and nitrogen (N) from the flue gas after combustion in oxygen at 950 °C. The size of a single sample was approximately 0.10 g with at least five repetitions for each material. The elemental standards for the LECO device used in calibration were: EDTA (ethylenediaminetetraacetic acid), rice flour, and rye flour. The measurements were conducted in a sulphur module of CHN628, using approximately 0.2 g of samples combusted in oxygen at 1350 °C. Oxygen was determined as a difference from 100% and the analysis results were converted according to ISO 16993 [51].

The higher heating value of the samples was determined using a LECO AC600 (LECO Corporation, USA) isoperibol bomb calorimeter. For each measurement, an approximately 1 g sample was pressed into a pellet and combusted in a calorimeter bomb filled with oxygen to 3 MPa. The reference temperature was 28 °C. The analyser was calibrated on benzoic acid. Conversions to sulphuric and nitric acids were not carried out. Otherwise, the method and lower heating value calculations were conducted according to ISO 1928 [52]. To compare the thermally treated samples, theoretical stoichiometrical combustion analysis was carried out. Stoichiometry is important to determine particular theoretical emission parameters of the biochars [53].

The chlorine content of the samples was not evaluated. On the one hand, the amount of chlorine affects the operation of corrosion [5,54] and, on the other hand, is the cause of the formation of dioxins (Lee et al., 2004). In, e.g., straw biomass, the chlorine content can be around 0.1% wt. [55,56]; however, in spruce wood, the chlorine content tends to be around 0.01% wt. [55]. Therefore, the amount of chlorine in spruce wood was considered negligible for stoichiometric calculations and would have no significant effect on the energy utilization of this type of biomass [57].

To illustrate the expected behaviour of the fuels in combustion and to compare them against average-quality coal, theoretical combustion analysis was carried out based on stoichiometric combustion equations. The methodology and equations are shown in, for example, Jeníček et al. [21].

### 2.2. Economic Evaluation of the Substitution of Solid Fossil Fuels with Wood Torrefaction Products

The torrefaction product was considered as a fuel alternative in a model CHP plant with a thermal output of 40 MW_th_ and a power output of 4 MW_e_ that combusts brown coal. The total steam output of the CHP plant was 61 t h^−1^ of superheated steam with a pressure of 1.4 MPa and temperature of 260 °C. The CHP plant was a district heating system in a municipality with 50 thousand inhabitants located in the CEE region, at 48° latitude. The CHP was located in temperate climate, with a mean annual temperature of 8.24 °C.

From an economic perspective, we compared two alternatives: A0, i.e., the current state without conversion, so the CHP plant would continue burning brown coal for heat and power production. The alternative, variant A1, i.e., the investment alternative that would use wood biochar torrefied at 350 °C. The hypothetical investment includes the modification of the CHP plant to enable production of torrefied wood and its subsequent energy use. Given the state of the CHP plant, infrastructure, facilities for biomass preparation, low-temperature dryer of input material, high-temperature dryer, torrefaction reactor, steam boiler for wood chip drying, and torrefaction product storage facilities needed to be installed. To determine the capital expenditures (*CAPEX*), figures according to Svanberg et al. [58] were used, scaled to the CHP size according to Equation (1). The scale factor used was 0.7, according to a recommendation in [58], because the CHP already had infrastructure partially installed for the needs of using coal as a fuel.

(1) $${COST}_{SIZE2}={COST}_{SIZE1}\;\times \;{\left(\frac{SIZE2}{SIZE1}\right)}^{scale\;factor}$$

In the financial and economic assessment, the *CAPEX* and operational costs, revenues, and cash flow generated by the two alternatives considered were observed. Economic analyses were carried out for a 15-year operation period, typical for this kind of investment in the Czech Republic, with the initial year of investment being 2023. The investment was assessed in fixed prices, i.e., without the effects of inflation. Only eligible costs defined as inseparable for heat and power production were considered, in the following structure: materials, depreciation and amortization, personnel costs, services, and financial costs. Material costs consisted of fuel costs (A0—brown coal at a price of 21.81 EUR per MWh^−1^ and A1—torrefied wood at a price of 34.97 EUR per MWh^−1^), water, residue disposal, consumed energies. Fuel was the main variable cost. Depreciation and amortization were included as tax depreciation, according to current Czech legislation. Personnel costs included wages, social security, and health insurance of the CHP personnel according to current Czech legislation. In A1, they also included the personnel costs of torrefaction facility personnel. Services included repairs and maintenance carried out by contractors. Financial costs for A1 included insurance (0.05%) and interest (5% p.a.). The tax rate used was 21%, according to current Czech legislation. The discount rate used was 2.5%, typical for this kind of investments in the Czech Republic. Carbon emission allowances were considered for A0, with a mean annual price of the European Union Allowance (EUA) applied as cost per ton of CO_2_ emitted–79.78 EUR per tCO_2_^−1^.

Further considerations:

- Revenues of the project consisted of supplied heat (price: 139.38 EUR per MWh^−1^) and electricity (price: 90.86 EUR per MWh^−1^).
- Revenues were calculated in fixed prices without value-added tax and other indirect taxes.
- Cash inflow did not include non-monetary revenues.

The feasibility of the alternatives was assessed based on the outcomes of dynamic methods of investment evaluation. They were the following: (i) net present value of the investment (*NPV*; Equation (2)), discounted payback period (*DPP*; Equation (3)), internal rate of return (*IRR*; Equation (4)), a profitability index (*PI*).

(2) $$NPV=\sum_{n=0}^{N}\frac{C_{n}}{{\left(1+r\right)}^{n}}-CAPEX$$

*n* is the year from the start of the investment until year 15;
*C_n_* net value of the cash flow in a given year;
*r* discount rate.

(3) $$DPP=y+\frac{abs\left(n\right)}{p}$$

*y* is the period preceding the period in which the cumulative cash flow turns positive;
*p* discounted value of the cash flow of the period in which the cumulative cash flow turns positive;
*abs* (*n*) absolute value of the cumulative discounted cash flow in period *y.*

(4) $$NPV=\sum_{n=0}^{N}\frac{C_{n}}{{\left(1+IRR\right)}^{n}}=0$$

*n* is the year from the start of the investment until year 15;
*C_n_* net value of the cash flow in a given year;
*IRR* internal rate of return.

## 3. Results and Discussion

### 3.1. Quality Parameters of the Solid Torrefaction Products

**Figure 1:**
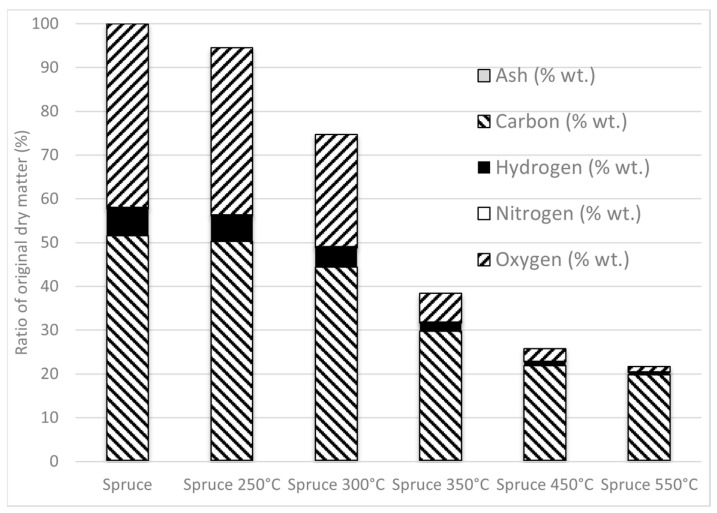
Mass yield of spruce biochar, heights of bars represent percentage of original dry matter.

The results of the elemental analyses showed a positive effect of the treatment process temperature on the wood samples. An accompanying advantage of thermal treatment is the reduction in water content, also resulting in an increase in calorific values. The oxygen and hydrogen concentrations in the samples also decreased proportionally with the increasing process temperatures [17]. Only a slight decrease was recorded at low temperatures, while Vassilev et al. [55] stated that, for phytomass, the oxygen content decrease is substantial. With the gradual increase in the process temperatures from 300 to 350 °C, the oxygen reduction in the spruce samples was considerable. Indeed, temperatures between 300 and 350 °C represent the upper threshold for torrefaction treatment [17]. Exceeding these temperature does not result in a considerable oxygen content reduction and lower dry mass yield (Figure 1), while increasing the yields of gaseous and liquid products [59,60]. The total dry mass yield at this threshold, i.e., at 350 °C, was 38.4%, with a net calorific value of 27.87 MJ kg^−1^, a result observed for other woody biomasses by Ingram et al. [61] or Jeníček et al. [62].

The samples of damaged spruce wood had a low ash content in their fresh state (Table 1), which is positive for the higher heating value of a fuel [38]. The fact that ash content did not exceed one percent content in the dry mass of the samples was positive, since it enables the use of the fuel in small-scale units [57,63] and gasification units [40]. The thermal treatment increased the amount of ash severalfold over the original spruce wood samples. However, compared to the reference, brown coal, or phytomass fuels, it was still very low [64,65].

Fuel sulphur concentration, which leads to the emission of sulphur oxides is usually a problem [66]; however, the spruce wood contained very low concentrations of sulphur, thus they were not considered in the study further.

The most substantial change of the parameters of the samples, when processed into biochar via torrefaction was observed between 300 and 350 °C. The biochar created at a temperature of 550 °C reached more than 90% wt. share of carbon and a lower than 5% wt. content of oxygen, while the share of hydrogen decreased from 5 to 3% at 550 °C. Generally, at higher temperatures, the sum of carbon and hydrogen content should increase to almost 100% wt., while oxygen should reduce to practically 0% wt. [17]. Carbon, oxygen, and hydrogen contents develop quite homogenously with increasing temperatures in woody biomass, regardless of the tree species or process conditions [67]. Increasing process temperatures resulted in a slight increase in the nitrogen content of the products. The relative increase was likely caused by the decreased share of other components during the thermal treatment, as has been the case for other types of input materials [60,68,69].

The input for torrefaction was considered to be pre-dried to 10% wt. moisture content, with a mass flow as shown in Table 2. The energy consumption for the processing of wood was calculated based on the heat capacity of biomass. The difference in heat capacity (J kg^−1^ K^−1^) compared to other types of biomasses is considerable [70,71]. About 1000 J kg^−1^ K^−1^ heat capacity were measured for biochars regardless of the treatment temperature [70]. Average heat capacity was estimated via a linear regression based on the measured values for spruce [70], reaching about 1550 J kg^−1^ K^−1^. Another important fact is that the form of carbon does not affect heat capacity [70]. For the treatment temperature selected for further consideration in the A1 variant (350 °C), the energy consumption for biochar production with a mass flow of 2345 kg h^−1^, which would be able to supply a 10 MW furnace, would be 345 kW, resp. 1381 kW for the substitution of 40 MW at a mass flow of 9383 kg h^−1^. For more precise measurements, total heat losses need to be considered that could only be obtained in real-life operation of a particular torrefaction reactor.

Within the analysis, the use of torrefaction by-products was considered—liquid and gaseous products that can be used for direct combustion, thus decreasing the amount of external energy needed to reach the temperature of 350 °C. Mass yield of biochar after torrefaction is depicted in Figure 1. It can be seen that most changes happen in the temperature range between 300 and 350 °C, where almost all parameters are subject to substantial change, mainly due to (almost complete) breakdown of hemicellulose, and partial breakdown of cellulose and lignin [17]; because of the lower structural stability of hemicellulose and cellulose, they exhibit a more rapid thermal breakdown. The total mass yield of dry matter at 350 °C was 38.4% of the initial dry mass, with the remainder of the input material leaving in liquid or gaseous form. The breakdown of hemicellulose and cellulose at higher temperatures increases the amount of condensable gases, while lignin, which is more stable, increases biochar yield [72].

To determine the theoretical heat consumption for torrefaction, the mass yield of biochar can be used, as shown in Figure 1. The torrefaction heat consumption of 9383 kg h^−1^ was based on several variables—mean ambient temperature at the proposed location (8.241 °C), the specific heat capacity of spruce wood (1,55 kJ kg^−1^ K^−1^), and input mass flow at 10% moisture content and lower heating value of 17.05 MJ kg^−1^. The total heat consumption for torrefaction would be 1656.8 kW at 80% heating efficiency. The theoretical net calorific value of torrefaction by-products was determined based on the elemental composition of the released matter, which made up 61.6% of the weight of the original raw material at the process temperature 350 °C. The torrefaction by-products were 21.8% carbon, 4.3% hydrogen, and 0.2% nitrogen and oxygen. The derived calorific value was around 5.5 MJ/kg, and since carbon will not be free and will bind to oxygen and hydrogen, the calorific value will be low, similar to the results of, e.g., Tabakaev et al. [73]. At 80% combustion efficiency, 1962 kWh of heat is generated, which was considered for use in heating the input material in the torrefaction reactor. Similar outcomes were observed by Cong et al. [74], who stated that 82.1% of the energy efficiency of a polygeneration system can be achieved.

**Figure 2:**
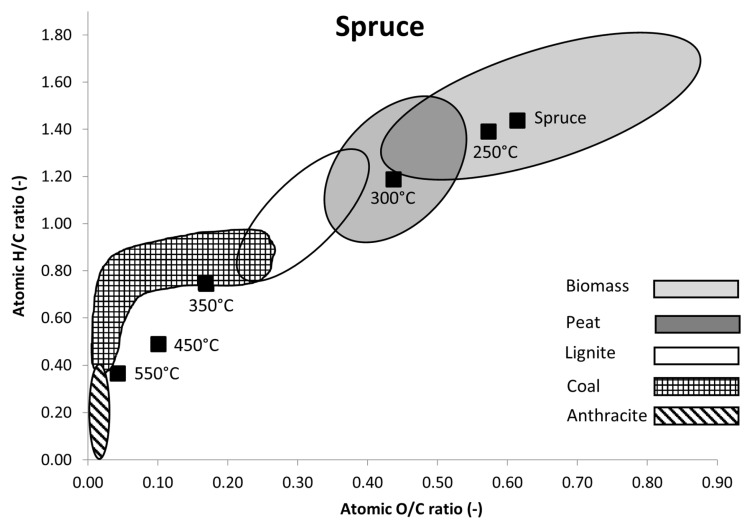
Van Krevelen diagram for spruce samples, ranges for material types according to Van Loo and Koppejan [75].

In a van Krevelen diagram (Figure 2), which shows the development of the three main fuel components—carbon, oxygen, and hydrogen—during carbonization, oxygen is released approximately twice as quickly as hydrogen, until hard coal forms. Further development to anthracite is usually accompanied by a decrease in the H/C ratio with a practically constant low oxygen content. Figure 1 shows the van Krevelen diagram for the biochar produced from spruce wood, where it can be seen that the increase in process temperatures lead to decreasing the H/C and O/C ratios. Similarly to a van Krevelen diagram for fossil fuels, the O/C ratio decreases at a higher rate than the H/C ratio. Based on the measurements, temperatures in the range between 300 and 350 °C lead to the largest decrease in the atomic ratios. A 100 °C temperature change decreased the O/C ratio from 0.75 to 0.49. A further increase of 100 °C decreases the O/C ration from 0.49 to 0.37. Similar decreases were observed for the H/C ratios—a temperature increase from 250 to 350 °C caused a decrease in the ratio from 1.5 to 0.8, a result similar to those observed for other woody biomasses [17].

### 3.2. Feasibility of Substituting Coal with Torrefied Wood as a CHP Plant Fuel

In the A1 alternative model, the substitution of brown coal with torrefied wood induced a capital expenditure (*CAPEX*) of 15.73 million EUR. Considering the facilities and infrastructure that were already installed in the original CHP plant for coal processing, the highest *CAPEX* was needed for the construction and installation of facilities for handling raw woody biomass, followed by the torrefaction reactor (Table 3). This *CAPEX* was covered by an investment loan with an annual instalment of 1.05 million EUR and an interest of 787 thousand EUR in the first year. Regarding the operational costs of the alternatives A0 and A1, Table 4 shows that in the non-investment alternative, the highest costs were those covering the purchase of carbon credits; more than fuel or any other cost type. Conversely, fuel costs for biochar production were the highest in the alternative A1 model. Since biochar is made from renewable biological resources, it was not necessary to consider carbon credits for the emissions resulting from their use. In both alternatives, other costs were relatively marginal, and had limited effects on the earnings before interest, taxes, depreciation, and amortization (*EBITDA*) or cash flows. Besides the financial costs—interest and insurance and personnel costs were also higher in the alternative A1 model than in A0, due to the staff needed for the operation of the torrefaction facilities.

Considering the *CAPEX*, torrefaction technology, as modelled in A1, was less expensive than the reconstruction of a coal-fired CHP to raw biomass (co)firing would be. Based on data provided by the NERA economic consultancy [76], the reconstruction of a CHP that enables raw biomass firing costs 33.6 million EUR, scaled to the size of the considered CHP plant. In contrast, the *CAPEX* for the construction and installation of the torrefaction facilities was estimated at 47% of the *CAPEX* needed for reconstruction to raw biomass firing. Furthermore, a conservative estimate of torrefaction *CAPEX* has been used [58], though the literature provides lower figures when scaled to the size of the considered CHP plant [77,78,79,80].

From the revenue perspective, the study was set up so that in both alternatives, the CHP plant would produce and supply the same amount of heat and power, with the same prices, i.e., no subsidy scheme was considered for supplying energies from renewable resources to the grid or district heating system. The CHP plant was used as a heat source for the nearby town, supplying heat to residential areas and industry, power was supplied to the national grid at commodity prices. Therefore, in both alternatives, annual revenues reached 15.749 million EUR for heat and 2.091 million EUR for power (Table 5).

**Figure 3:**
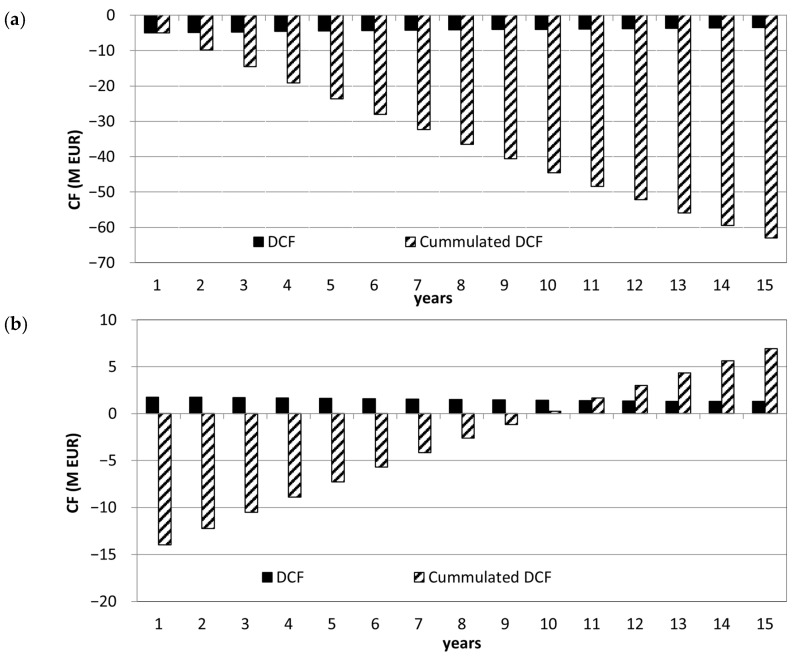
Development of annual discounted cash flow (DCF) and cumulated discounted cash flow (cumulated DCF) in (**a**) the A0 alternative and (**b**) the A1 alternative.

Table 6 shows that based on basic financial parameters, using biochar as fuel is feasible and more profitable in current conditions than using brown coal. The considerably higher fuel costs of biochar were compensated by not needing to purchase carbon credits (Table 4). The A1 investment alternative generated a positive *EBITDA* of 2.32 million EUR and a net cash flow of 1.82 million EUR. The cumulated discounted cash flow reached 22.67 million EUR in the observed period. When looking at the dynamic investment appraisal, the *NPV* reached 6.94 million EUR in A1 and −63 million EUR in A0. The profitability index of A1 was 1.44, and its *IRR* was 7.92%. The development of discounted cash flow and its cumulation in Figure 3 shows that A1 reaches overall positive financial flows after about ten years of operation. Considering that A1 reached positive cash flows and *NPV* at fuel prices 1.6-times higher than A0, we can consider it a feasible investment. Even if the price of the biochar increased from 34.97 to 36.74 EUR t^−1^, A1 would still reach a slightly positive *NPV* and a discounted payback period of approximately 15 years.

## 4. Conclusions

The results showed that torrefaction can yield high-grade biochar suitable for substitution of fossil fuels. Foremost, the lower heating value of dry matter of biochar processed at 350 °C substantially exceeds that of brown coal. Another considerable benefit of using biochar for energy is its lower ash content compared to coal, resulting in savings in residue handling costs. Low concentrations of sulphur and nitrogen in the fuel and flue gases, which again shows environmental benefits of using it as fuel instead of coal. Economic assessment too, yielded encouraging results, showing that constructing torrefaction facilities is less intensive on the capital expenses compared to raw biomass firing, while generating positive cash flows because biochar is considered a CO_2_-neutral fuel. The study therefore provides a feasible avenue for decarbonizing the energy sector, one of the main industries contributing to carbon emissions of the EU, as required by the EU policy (The European Green Deal 2019). As shown in this study, low-grade timber from salvage logging and other forest-based wood streams can serve as inputs into biochar production, thus helping decarbonize the energy sector partially. This is supported also by the goals of the Directive on renewable energy sources (2021), where it is stated that increasing the share of energy supplied from renewable energy sources is vital for the EU economy. The current goal stated in the Directive for the year 2030 is a 32% share of renewables, though the revised proposal of the European Commission counts on at least a 40% share on the EU energy market until 2030 (Fit for 55, 2021). The processing of low-grade wood from salvage logging into a stable and energy-rich fuel in the form of biochar can secure stable energy supply in the EU at accessible prices and can also be used as a unique solution to sequester energy and carbon, while enabling the development of an integrated and interconnected energy market.

## Figures and Tables

**Table 1:** Proximal and ultimate analysis of spruce and biochar samples (a.r.—as received, sample with a natural amount of moisture, d.b.—on dry basis).

Sample	Water Content (% wt.)	Ash (% wt.)	Carbon (% wt.)	Hydrogen (% wt.)	Sulphur (% wt.)	Nitrogen (% wt.)	Oxygen (% wt.)	Gross Calorific Value (MJ kg^−1^)	Net Calorific Value (MJ kg^−1^)
	*W*	*A*	*C*	*H*	*S*	*N*	*O*	*Q_s_*	*Q_i_*
Coal a.r.	39.50	10.28	41.00	3.10	0.61	0.60	4.91	15.64	14.00
Coal d.b.		17.00	67.77	5.12	1.00	0.99	8.12	24.26	23.14
Spruce a.r.	40.00	0.17	30.75	3.71	0.00	0.21	25.16	12.07	10.28
Spruce d.b.		0.28	51.25	6.19	0.00	0.35	41.93	20.12	18.77
Spruce 250 °C a.r.	3.00	0.30	51.29	5.99	0.00	0.30	39.12	20.18	18.80
Spruce 250 °C d.b.		0.31	52.88	6.17	0.00	0.31	40.33	20.81	19.46
Spruce 300 °C a.r.	3.00	0.39	57.27	5.71	0.00	0.32	33.31	21.83	20.51
Spruce 300 °C d.b.		0.41	59.04	5.89	0.00	0.33	34.33	22.51	21.22
Spruce 350 °C a.r.	3.00	0.75	74.42	4.66	0.00	0.44	16.73	28.05	26.96
Spruce 350 °C d.b.		0.78	76.72	4.80	0.00	0.45	17.25	28.92	27.87
Spruce 450 °C a.r.	3.00	1.11	81.20	3.34	0.00	0.48	10.87	29.50	28.70
Spruce 450 °C d.b.		1.14	83.71	3.44	0.00	0.49	11.22	30.41	29.66
Spruce 550 °C a.r.	3.00	1.32	87.50	2.69	0.00	0.57	4.92	31.25	30.59
Spruce 550 °C d.b.		1.36	90.21	2.77	0.00	0.59	5.07	32.21	31.61

**Table 2:** Combustion analysis using stoichiometric calculations (a.r.—sample as received, respectively, with the expected amount of moisture).

		Coal a.r.	Spruce a.r.	Spruce 250 °C a.r.	Spruce 300 °C a.r.	Spruce 350 °C a.r.	Spruce 450 °C a.r.	Spruce 550 °C a.r.
Fuel mass flow into boiler with a 90% efficiency rate and 10 MW thermal output	kg h^−1^	2857	3889	2128	1950	1484	1394	1308
Fuel mass flow into boiler with a 90% efficiency rate and 40 MW thermal output	kg h^−1^	11,428	15556	8510	7800	5935	5575	5231
Theoretical oxygen flow for complete combustion (*n* = 1)	kg kg^−1^	1.298	0.865	1.456	1.651	2.190	2.324	2.499
Theoretical air flow for complete combustion (*n* = 1)	kg kg^−1^	5.596	3.730	6.274	7.116	9.440	10.015	10.773
Mass of air for complete combustion (*n* = 2.1)	kg kg^−1^	11.752	7.834	13.176	14.944	19.824	21.032	22.622
Mass of humid flue gas (*n* = 2.1)	kg kg^−1^	12.984	9.055	14.548	16.366	21.381	22.620	24.253
Mass of dry flue gas (*n* = 2.1)	kg kg^−1^	11.839	8.008	13.452	15.224	20.139	21.448	23.077
Theoretical mass of dry flue gas (*n* = 1)	kg kg^−1^	5.745	3.945	6.619	7.474	9.858	10.541	11.345
Mass quantity of CO_2_ (*n* = 2.1)	kg kg^−1^	1.509	1.131	1.887	2.107	2.738	2.987	3.219
Mass of SO_2_ (*n* = 2.1)	kg kg^−1^	0.012	0.00	0.00	0.00	0.00	0.00	0.00
Mass of N_2_ (*n* = 2.1)	kg kg^−1^	8.875	5.915	9.947	11.282	14.966	15.879	17.080
Mass of O_2_ (*n* = 2.1)	kg kg^−1^	1.428	0.952	1.601	1.816	2.409	2.556	2.749

**Table 3:** Capital expenditures (*CAPEX*) needed for construction and installation of the torrefaction facilities in A1 variant.

CAPEX Item	Value (Millions of EUR)	Value Scaled (Millions of EUR)
Infrastructure and buildings	5.95	2.32
Tipping bunkers and biomass processing	6.55	2.55
Low-temperature drier	2.27	0.88
High-temperature steam drier	4.41	1.72
Torrefaction reactor	5.51	2.15
Steam boiler	11.1	4.32
Product cooling	1.12	0.44
Milling	0.27	0.11
Discharging and outdoor storage	3.22	1.25
Total	40.4	15.73
Interest on capital	-	0.787
Instalments payment	-	1.05

**Table 4:** Operational costs in both alternatives.

Operational Cost Item (Millions of EUR)	A0	A1
Fuel costs	8.497	11.661
Carbon permits costs	12.048	0
Material costs	1.261	0.462
Services	0.093	0.093
Personnel costs	1.029	1.338
Amortization and depreciation	0	0.865
Interest	0	0.787
Insurance (related to investment)	0	0.079
Costs without depreciation	22.928	14.419
Costs without depreciation and interest	22.928	16.390
Total costs	22.928	15.284

**Table 5:** Revenues for heat and power supply as generated by the CHP plant in both alternatives.

Revenue Item	A0 and A1
Heat supplied (MWh year^−1^)	112,995
Mean heat price (EUR per MWh^−1^)	139.38
Revenues for heat supply (millions of EUR per year^−1^)	382.79
Electricity supplied (MWh per year^−1^)	29,011
Electricity price (EUR per MWh^−1^)	90.86
Revenues for electricity supply (millions of EUR per year^−1^)	50.82
Total revenues (millions of EUR per year^−1^)	433.61

**Table 6:** Cash flow of the alternatives in the first year of operation of the CHP plant.

Cash Flow Item (Millions of EUR)	A0	A1
Revenues	17.839	17.839
Costs	−22.928	−16.212
EBITDA	−5.088	1.628
Depreciation and amortization	0	−0.036
EBIT	−5.088	1.592
Interest	0	−0.032
EBT	−5.088	1.560
Taxes	0	−0.296
Net profit	−5.088	1.263
Cash flow	−5.088	1.299
Net cash flow	−5.088	1.256
Discounted cash flow	−4.964	1.225

## Data Availability

The datasets used or analysed during the current study are available from the corresponding author on reasonable request.
